# mTORC1 Regulates Flagellin-Induced Inflammatory Response in Macrophages

**DOI:** 10.1371/journal.pone.0125910

**Published:** 2015-05-05

**Authors:** Wenlei Bao, Yanfeng Wang, Yuting Fu, Xiaoyang Jia, Jiaxin Li, Nyamtsengel Vangan, Lili Bao, Huifang Hao, Zhigang Wang

**Affiliations:** 1 College of Life Science, Inner Mongolia University, Hohhot, China; 2 College of Basic Medical Science, Inner Mongolia Medical University, Hohhot, China; H.Lee Moffitt Cancer Center & Research Institute, UNITED STATES

## Abstract

Bacterial flagellin triggers inflammatory responses. Phosphoinositide 3-kinase (PI3K) and mammalian target of rapamycin (mTOR) regulate the production of pro- and anti-inflammatory cytokines that are induced by extrinsic antigens, but the function of mTORC1 in flagellin-induced inflammatory response is unknown. The purpose of this study was to examine the role and the mechanism of PI3K/Akt/mTOR pathway in flagellin-induced cytokine expression in mouse macrophages. We observed that flagellin upregulated TNF-α time- and dose-dependently. Flagellin stimulated rapid (<15 min) PI3K/Akt/mTOR phosphorylation that was mediated by TLR5. Inhibition of PI3K with LY294002 and wortmannin, and of mTORC1 with rapamycin decreased flagellin-induced TNF-α and IL-6 expression and cell proliferation. The activation of NF-κB p65 and STAT3 was regulated by mTORC1 via degradation of IκBα and phosphorylation of STAT3 in response to flagellin, respectively. Thus, the PI3K/Akt/mTORC1 pathway regulates the innate immune response to bacterial flagellin. Rapamycin is potential therapy that can regulate host defense against pathogenic infections.

## Introduction

Flagellin is a pathogen-associated molecular pattern (PAMP) that is recognized by pattern recognition receptors, resulting in innate immune responses in diverse organisms, including flies, plants, and mammals [1–3]. Extracellular and cytoplasmic bacterial flagellin induces immune responses. Mediated by TLR5, extracellular flagellin effects inflammatory gene expression, in intestinal epithelia and promonocytic cells [1,4–7], in which entails the release of proinflammatory cytokines, such as TNF-α and IL-6 via NF-κB activation [8,9]. TNF-α is a proinflammatory cytokine that regulates the immuno-inflammatory response [10]. IL-6 is a multifunctional cytokine that controls immune responses, inflammation, hematopoiesis, bone metabolism, and immunity [11,12] and is involved in the pathogenesis of autoimmune diseases and chronic inflammation [13]. Conversely, cytoplasmic flagellin activates caspase-1 and induces the secretion of IL-1β through IPAF, a cytosolic pattern recognition receptor [14,15]. The inflammasome is activated by cytoplasmic flagellin via NLR apoptosis inhibitory protein 5 (Naip5) [16]. The inflammasome is a large cytoplasmic multiprotein complex, that effects the secretion of IL-1β [17,18], a proinflammatory cytokine that protects the host from infection [19].

Phosphoinositide 3-kinase (PI3K) is a phosphatidylinositol kinase that regulates innate immune responses that are induced by bacterial components, such as CpG DNA [20], LPS [21], flagellin [22], and byproducts of viral infections [23], but has the double-edged function in TLR-mediated inflammatory cytokine expression. Several reports have demonstrated that PI3K is proinflammatory [21,24,25]. In contrast, PI3K negatively regulates synthesis of the proinflammatory cytokine IL-12 in DCs [26], and PI3K activation limits IL-6 and IL-8 expression in epithelial cells [22]. PI3K leads to PIP2 to PIP3; subsequently, PIP3 activates Akt [27,28]. LY294002, a PI3K inhibitor, decreases the phosphorylation and kinase activity of Akt (Ser473) [29].

Mammalian target of rapamycin (mTOR) is a conserved serine/threonine kinase that controls transcription, translation, cell proliferation, and apoptosis. mTOR forms 2 distinct complexes with other proteins: mTOR complex 1 (mTORC1) and mTOR complex 2 (mTORC2); only mTORC1 is rapamycin-sensitive. The mTOR signaling pathway lies downstream of PI3K [30], of which ribosomal p70S6 kinase (p70S6K) and initiation factor 4E-binding protein 1 (4EBP-1) are downstream effectors [31]. mTORC1 signaling regulates LPS-induced pro- and anti-inflammatory cytokine production in various cells, such as macrophages, monocytes, DCs, and other immune cells [10,32–35]. Moreover, it regulates viral dsRNA-induced inflammatory responses in keratinocytes [36]. However, the functions of mTORC1 and PI3K/Akt/mTOR signaling in flagellin-induced inflammatory response are unknown.

In this study, we treated mouse macrophages with LY294002 and measured proinflammatory gene expression. We also determine the effects of rapamycin, a specific inhibitor of mTOR, on flagellin-induced proinflammatory gene expression and activation of transcription factors. Our data suggest that PI3K/Akt/mTOR signaling mediates flagellin-induced proinflammatory gene expression via TLR5-dependent mechanism and that the mTOR pathway regulates NF-κB and STAT3 activation to regulate gene expression in response to flagellin in mouse macrophages.

## Materials and Methods

### Ethics statement

The mouse procedure used in this study is approved by the Inner Mongolia University Animal Care and Use Committee.

### Cell culture conditions

The Ana-1 mouse macrophage cell line was purchase from Cell Bank of Chinese Academy of Sciences. The cell line was cultured in complete medium (RPMI 1640, containing 10% heat-inactivated FCS, 2 mM L-glutamine, 100 U/ml penicillin, and 100 mg/ml streptomycin) at 37°C in a humidified atmosphere with 5% CO_2_. Mouse peritoneal macrophages were isolated 5 min after injecting RPMI 1640 medium without fetal bovine serum into the peritoneal cavity of ICR mouse. The cells were seeded in RPMI 1640 medium supplemented with 100 U/ml penicillin, 100 mg/ml streptomycin, and 10% fetal bovine serum and incubated in 5% CO_2_ at 37°C.

### Reagents and antibodies

Purified flagellin from *S*.*typhimurium* ligand (ultrapure) TLR5 was purchased from InvivoGen (San Diego, CA, USA). Wortmannin was purchased from Sigma Chemical (St Louis, MO, USA). Rapamycin was purchased from Gene Operation (Ann Arbor, Michigan, USA). LY294002 was purchased from Cell Signaling (Beverley, MA, USA). Antibody to β-actin was purchased from Sigma Chemical. Antibodies to phospho-p44/42 MAPK (Erk1/2) (Thr202/Tyr204), p44/42 MAPK (Erk1/2), phospho-Akt (Ser473), Akt, phospho-S6 (Ser240/244), S6, phospho-4EBP1 (Thr37/46), IκBα, NF-κB p65, phospho-STAT3 (Tyr705), and STAT3 were purchased from Cell Signaling (Beverley, MA, USA). Antibodies to 4EBP1, phospho-mTOR (Ser2448), mTOR, TLR5, TLR4 and phospho-NF-κB p65 (Ser536) were purchased from Abcam (Cambridge, UK). Cytokine ELISA kits were obtained from eBioscience (San Diego, CA, USA).

### Cytokine ELISA

Cell culture supernatants were collected, centrifuged to remove cellular debris, and assayed immediately or stored at -80°C until analysis. Cytokines were measured per the ELISA kit manufacturer’s instructions, and absorbance was read at 450 nm and 570 nm on a Varioskan Flash Multimode Reader (Thermo Fisher Scientific, Pittsburgh, PA, USA). The detection limit of TNF-α and IL-6 was 3.9 pg/ml. All measurements were made in triplicate, and the mean values of the three independent measurements were used for the statistical analysis.

### Western blot analyses

Ana-1 cells were treated with flagellin in the presence or absence of LY294002 or rapamycin and washed two times with ice-cold PBS (pH 7.4). Cells were lysed in lysis buffer that contained 25 mM Tris-HCl (pH 7.6), 150 mM NaCl, 1% Nonidet P-40, 1% sodium deoxycholate, 0.1% SDS, protease inhibitor mixture, and phosphatase inhibitors (Sigma, Chemicals) and then placed on ice for 10 min. Next, the cells were harvested by scraping and centrifuged at 4°C for 10 min at 13,000 rpm.

Equal amounts of cell lysates were separated by SDS-PAGE on 10% polyacrylamide gels and transferred to PVDF membranes, which were then immunoblotted with the designated primary antibodies. The membranes were then treated with horseradish peroxidase-conjugated goat anti-rabbit IgG or goat anti-mouse IgG (GE Healthcare, UK) and detected using the ECL detection (Thermo Fisher Scientific, Pittsburgh, PA, USA) by exposure to X-ray film. The resolved bands were quantified using Gel-Pro Analyzer 4.0 (Media Cybernetics, USA).

### Cell viability assay

Ana-1 cells were used to seed 96-well plates at 1×10^3^ cells/well for culture. After 48 h, the cells were preincubated with LY294002 (10 μM), wortmannin (10 nM) or rapamycin (100 nM) 4 h before stimulation with flagellin (100 ng/ml) for 24 h, and cell proliferation was measured by methyl thiazolyl tetrazolium (MTT) assay. Briefly, 0.02 mL MTT solution (5 mg/mL in PBS) was added to each well and incubation for 4 h at 37°C, after which the media was replaced with 0.15 mL dimethyl sulfoxide, followed by a 10-min incubation. Then, the optical density was measured at 490 nm with a Varioskan Flash Multimode Reader (Thermo Fisher Scientific, Pittsburgh, PA, USA). All measurements were performed in sextuplicate, and the means of the three independent measurements were used for the statistical analysis.

### TLR5 blocking antibody assay

Ana-1 cells were used to seed 12-well plates in 1 ml medium per well in the presence or absence of TLR5 or TLR4 blocking antibody and stimulated with flagellin. After 24 h, cell culture supernatants were harvested, centrifuged to remove cellular debris, and assayed by ELISA. Cells were lysed, and total protein was collected for western blot analysis.

### Statistical analysis

The groups were compared by Fisher’s LSD or Bonferroni post-hoc analysis in one-way ANOVA using SPSS 19. The results are expressed as mean ± SD. Statistical significance was accepted at *p*< 0.05.

## Results

### Flagellin induces TNF-α expression in a time- and dose-dependent manner

**Fig 1:**
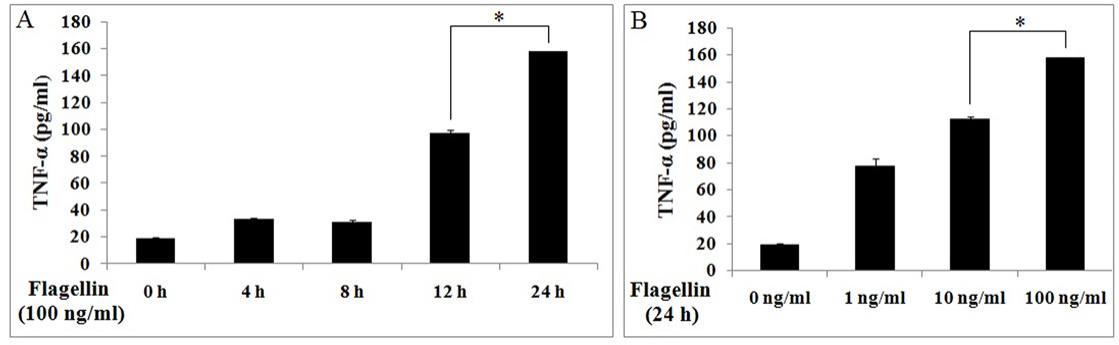
Flagellin induces TNF-α expression in a time-dependent and dose-dependent manner. Ana-1 cells were treated with flagellin, and supernatants were assayed for TNF-α by ELISA. A. flagellin (100 ng/ml) for 0, 4, 8, 12, and 24 h. B. flagellin (0, 1, 10, 100 ng/ml) for 24 h. Experiments were made in triplicate, and data are representative of three separate experiments (mean ± SD).

To determine the appropriate incubation time and dose of flagellin, we studied the effects of fiagellin on Ana-1 cells. Cells were stimulated with 0–100 ng/ml flagellin for 0–24 h, and TNF-α expression was measured by ELISA. TNF-α levels rose with the increasing incubation times and doses (Fig 1A and 1B), indicating that flagellin upregulates TNF-α time- and dose-dependently. TNF-α expression peaked at treatment with 100 ng/ml flagellin for 24 h, thus this condition was used for all subsequent experiments.

### LY294002, wortmannin and Rapamycin attenuate flagellin-induced TNF-α and IL-6 expression in Ana-1 cells

**Fig 2:**
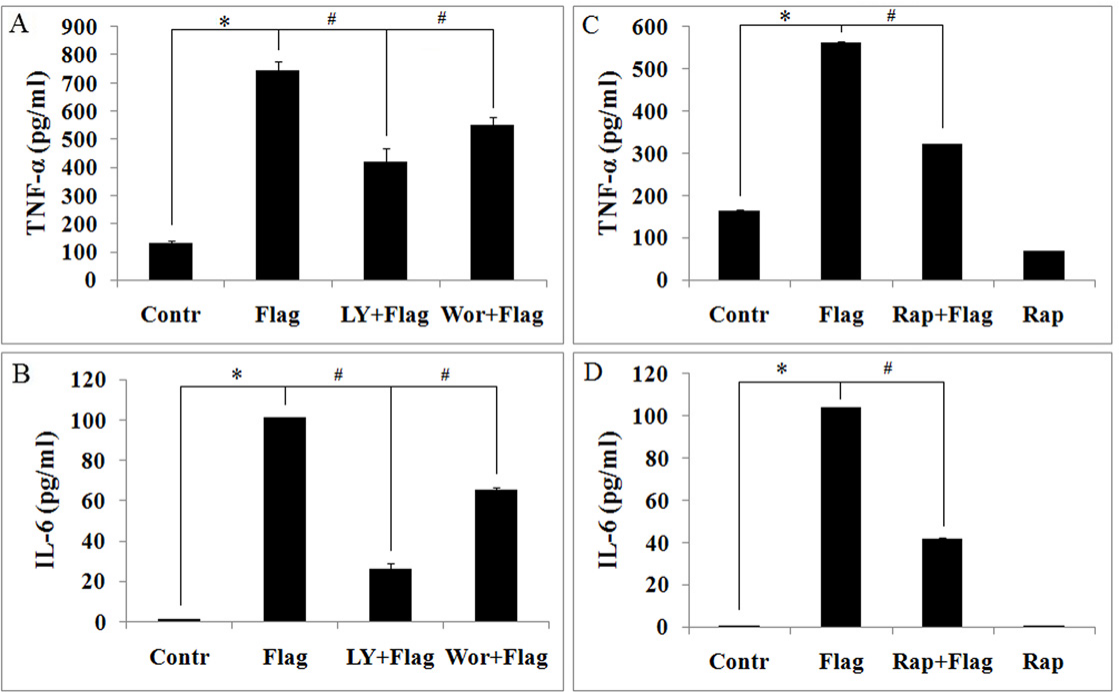
LY294002, wortmannin and rapamycin dowregulates flagellin-induced TNF-α and IL-6 expression in mouse macrophages. Ana-1 cells were pretreated with LY294002 (10 μM), wortmannin (10 nM) and rapamycin (100 nM) for 4 h before being stimulated with 100 ng/ml flagellin for 24 h. Supernatants were assayed for TNF-α and IL-6 by ELISA. A. flagellin-induced TNF-α is inhibited by LY294002 and wortmannin. B. flagellin-induced IL-6 expression is inhibited by LY294002 and wortmannin. C. flagellin-induced TNF-α is inhibited by rapamycin. D. flagellin-induced IL-6 expression is inhibited by rapamycin. Experiments were made in triplicate, and data are representative of three separate experiments (mean ± SD). *p< 0.05 compare to untreated controls, # *p*< 0.05 compared with flagellin group.

To determine whether PI3K regulates flagellin-induced expression of proinflammatory cytokines in mouse macrophages, we examined the effects of LY294002 and wortmannin on TNF-α and IL-6 expression in response to flagellin. The result shows that TNF-α and IL-6 levels in response to flagellin fell significantly due to LY294002 and wortmannin (Fig 2A and 2B), suggesting that PI3K/Akt regulates flagellin-induced proinflammatory cytokine expression.

To further examine the mechanism by which PI3K regulates flagellin-induced TNF-α and IL-6 expression, we measured the effects of mTOR on TNF-α and IL-6 levels in response to flagellin. TNF-α and IL-6 levels declined significantly due to rapamycin (Fig 2C and 2D), suggesting that mTOR controls flagellin-induced TNF-α and IL-6 expression in Ana-1 cells.

### LY294002, wortmannin and Rapamycin inhibit flagellin-induced Ana-1 cells proliferation

**Fig 3:**
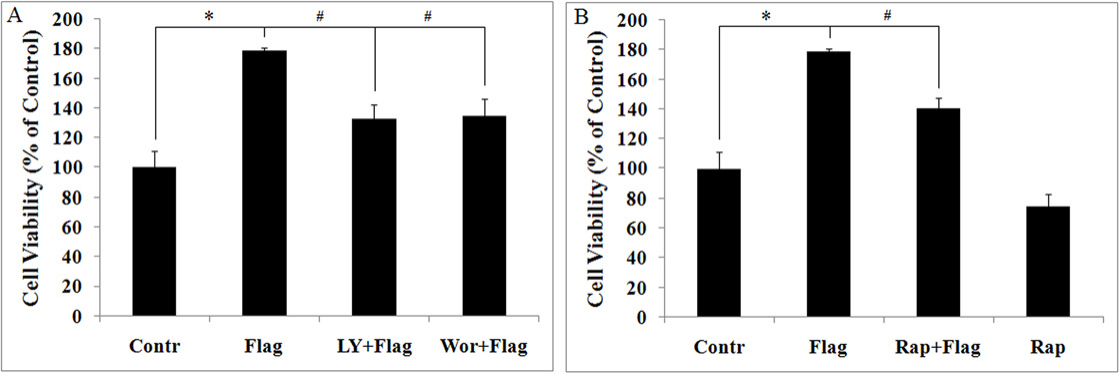
LY294002, wortmannin and rapamycin attenuates flagellin-induced cell proliferation. Ana-1 cells were pretreated with LY294002 (10 μM), wortmannin (10 nM) and rapamycin (100 nM) for 4 h and then stimulated with 100 ng/ml flagellin for 24 h, and cell proliferation was measured by methyl thiazolyl tetrazolium (MTT) assay. A. Proliferation was inhibited by LY294002 and wortmannin in response to flagellin. B. Proliferation was inhibited by rapamycin in response to flagellin. Experiments were made in triplicate, and data are representative of three separate experiments (mean ± SD). *p< 0.05 compare to untreated controls, # *p*< 0.05 compared with flagellin group.

To measure the effects of flagellin on mouse macrophages proliferation, Ana-1 cells were stimulated with 100 ng/ml flagellin for 24 h, and cell proliferation was measured by MTT assay. Flagellin enhanced Ana-1 cell proliferation, an effect that was inhibited by pretreatment with LY294002 (10 μM) and wortmannin (10 nM) for 4 h (Fig 3A). These results suggest that PI3K/Akt mediates flagellin-induced Ana-1 cell proliferation.

Next, we treated Ana-1 cells with rapamycin in the presence of flagellin to determine the influence of mTORC1 signaling on their proliferation. We found that flagellin-induced mTOR, 4EBP1, and S6 phosphorylation was inhibited by rapamycin (S1 Fig), indicating that rapamycin inhibits flagellin-induced mTOR signaling. Further, Ana-1 cell proliferation was suppressed by rapamycin in response to flagellin (Fig 3B). These results suggest that flagellin-induced proliferation is regulated by mTOR.

### Flagellin activates PI3K/Akt/mTOR signaling in Ana-1 cells via a TLR5-dependent mechanism

**Fig 4:**
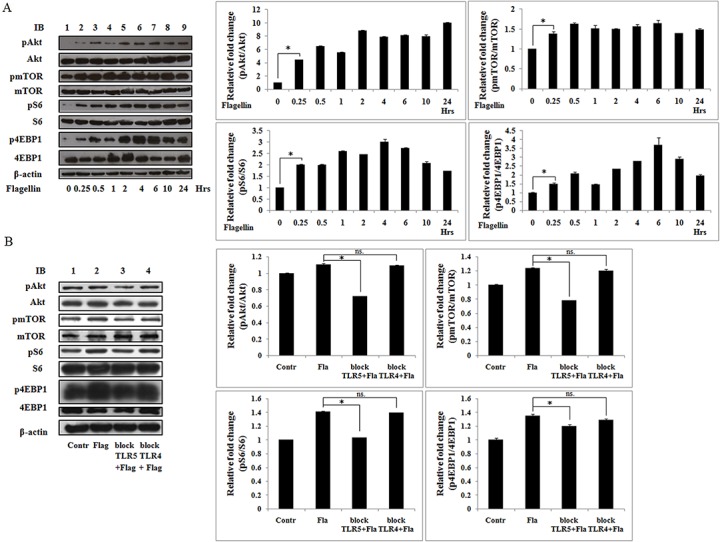
Flagellin activates PI3K/Akt/mTORC1 signaling via TLR5. Ana-1 cells were lysed, and total proteins were extracted. A. the cells were stimulated at the indicated times with *S*. *typhimurium* flagellin (100 ng/ml). B. the cells were stimulated by flagellin (100 ng/ml) for 24 h in present or absent of TLR5 or TLR4 antibody. Total proteins were subjected to immunoblot with phospho-Akt(Ser473), phospho-mTOR (Ser2448), phospho-S6 (Ser240/244), and phospho-4EBP1 (Thr37/46). Blots were stripped and probed with total Akt, mTOR, S6, and 4EBP1 antibodies. β-actin was used as a loading control. Three separate experiments were performed. *p< 0.05, ns. not significant.

Next, we examined the effects of flagellin on PI3K/Akt/mTORC1 signaling. Phospho-Akt and-mTOR levels increased significantly within 15 min, persisting for at least 24 h. Phospho-4EBP1 and-S6 rose within 15 min and peaked between 2 and 6 h, these levels were maintained for at least 24 h (Fig 4A). These results indicate that flagellin effects rapid activation of PI3K/Akt/mTOR signaling for at least 24 h in Ana-1 cells. To investigate whether flagellin activates PI3K/Akt/mTORC1 signaling via TLR5 in Ana-1 cells, we blocked activation of the small amount of endogenous TLR5 by TLR5 antibody, TLR4 antibody as a control. Increased phospho-Akt,-mTOR, -4EBP1 and-S6 levels were inhibited by TLR5 antibody, but not TLR4 (Fig 4B). Thus, flagellin induces PI3K/Akt/mTORC1 signaling in macrophages via a TLR5-dependent mechanism.

### PI3K mediates flagellin signaling to activate the Akt/mTORC1 pathway but not p44/42 MAPK (Erk1/2)

**Fig 5:**
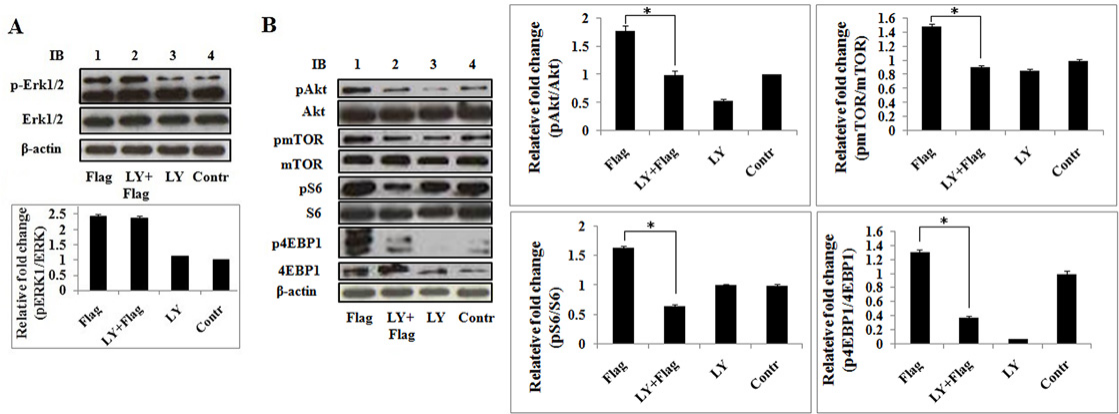
LY294002 decreases flagellin-induced Akt/mTOR activation but does not affect p44/42 MAPK (Erk1/2) activation. Ana-1 cells were pretreated with LY294002 (10 μM) for 4 h and stimulated with 100 ng/ml flagellin for 24 h. A. Total proteins were subjected to immunoblot with phospho-p44/42 MAPK (Erk1/2)(Thr202/Tyr204), the blot was stripped and probed with p44/42 MAPK (Erk1/2). B. Total proteins were subjected to immunoblot with phospho-Akt (Ser473), phospho-mTOR (Ser2448), phospho-S6 (Ser240/244), and phospho-4EBP1 (Thr37/46) antibodies. The blots were stripped and probed with Akt, mTOR, S6, and 4EBP antibodies. β-actin was used as the loading control. Three separate experiments were performed.

To identify the downstream targets of PI3K-mediated flagellin signaling, LY294002 was used to inhibit PI3K and examine the activation of flagellin-induced p44/42 MAPK (Erk1/2) and Akt/mTORC1 in Ana-1 cells. Phospho-p42/44 MAPK (Erk1/2),-Akt,-mTOR,-S6, and -4EBP1 were measured by immunoblotting after treatment with flagellin in the presence or absence of LY294002. Phospho-Erk1/2 levels increased significantly after stimulation with flagellin for 24 h, but LY294002 did not affect flagellin-induced phosphorylation of Erk1/2 (Fig 5A). However, the activation of Akt, mTOR, S6, and 4EBP1 by flagellin was attenuated by LY294002 (Fig 5B). These results indicate that PI3K governs flagellin-induced Akt/mTOR signaling but does not affect activation of p44/42 MAPK (Erk1/2) by flagellin. Thus, PI3K and Erk1/2 mediate flagellin signaling in parallel.

### Rapamycin impairs flagellin-induced NF-κB and STAT3 activation

**Fig 6:**
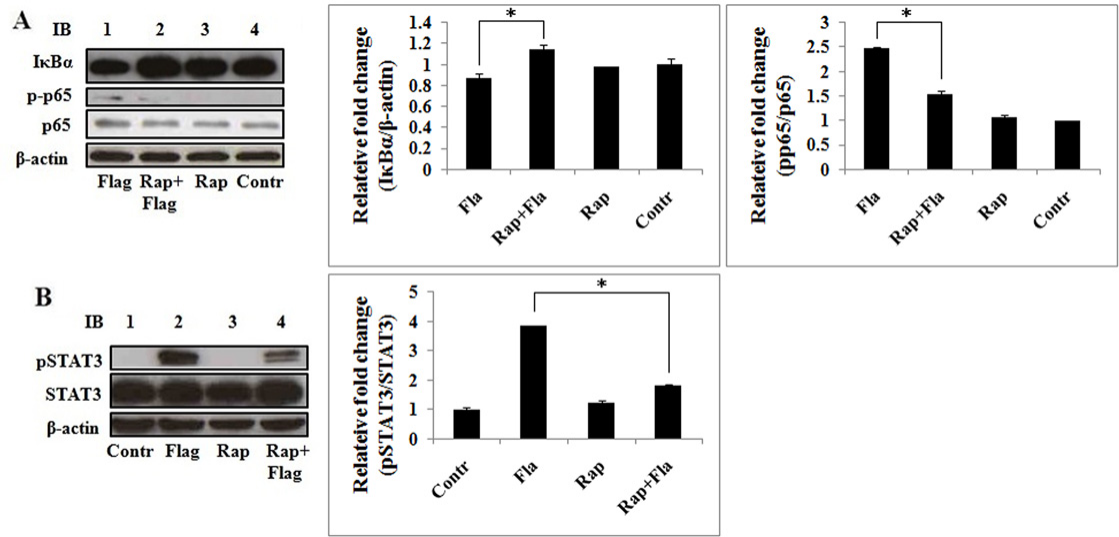
Rapamycin prevents flagellin-induced IκBα degradation and p65 and STAT3 activation. Ana-1 cells were pretreated with rapamycin (100 nM) for 4 h and stimulated with 100 ng/ml flagellin for 24 h. Total proteins were separated by SDS-PAGE and probed with IκBα, phospho-NF-κB p65 (Ser536), and phospho-STAT3 (Tyr705) antibodies. The blots were stripped and probed with NF-κB p65 and STAT3 antibodies. β-actin was used as the loading control. Three separate experiments were performed.

To determine the mechanism by which mTOR regulates proinflammatory cytokine gene expression, we studied the function of rapamycin in flagellin-induced activation of NF-κB and STAT3, two important transcription factors. Ana-1 cells were pretreated with or without 100 nM rapamycin before stimulation with flagellin, and IκBα, NF-κB, and STAT3 activation was detected by immunoblotting. Flagellin promoted IκBα degradation and NF-κB p65 phosphorylation, which were inhibited by rapamycin, indicating that mTOR regulates NF-κB p65 activation by degrading IκBα in response to flagellin (Fig 6A). Also, flagellin enhanced STAT3 phosphorylation, which was attenuated by rapamycin (Fig 6B). These data indicate that mTOR is central in the regulation of flagellin-induced NF-κB and STAT3 activation.

### TLR5 mediates flagellin-induced proinflammatory gene expression and STAT3 activation in Ana-1 cells

**Fig 7:**
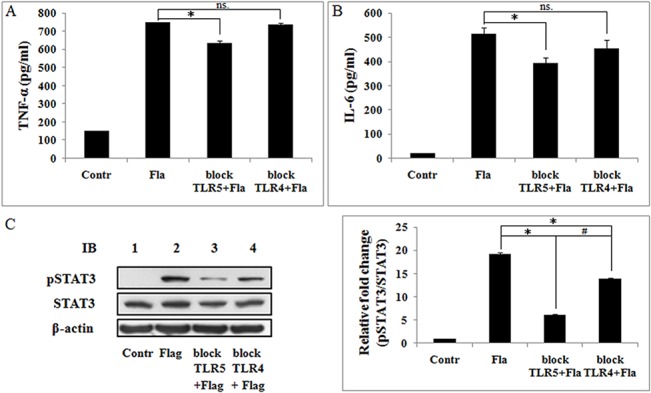
TLR5 mediates flagellin-induced TNF-α and IL-6 expression and STAT3 activation in macrophages. Ana-1 cells were pretreated with TLR5-blocking antibody or TLR4-blocking antibody for 24 h and stimulated with 100 ng/ml flagellin for 24 h. Supernatants were assayed for TNF-α and IL-6 by ELISA. A. Flagellin-induced TNF-α expression is inhibited by TLR5 blocking antibody. B. Flagellin-induced IL-6 expression is inhibited by TLR5 blocking antibody. Experiments were made in triplicate, and data are representative of three separate experiments (mean ± SD). *p< 0.05, ns. not significant. C. Total proteins were separated by SDS-PAGE and probed phospho-STAT3 (Tyr705) antibodies. The blots were stripped and probed with STAT3 antibodies. β-actin was used as the loading control. Three separate experiments were performed. *p< 0.05 compare to flagellin group, # *p*< 0.05 compared with TLR4 antibody group.

To determine whether TLR5 mediates flagellin-induced cytokine expression in Ana-1 cells, we measured flagellin-induced expression of TNF-α and IL-6 by ELISA, and STAT3 activation by western blot in the presence or absence of TLR5 blocking antibody, TLR4 antibody as a control. TNF-α and IL-6 levels declined significantly due to TLR5-blocking antibody, but not TLR4 (Fig 7A and 7B), and phospho-STAT3 levels of stimulation by flagellin were inhibited by TLR5-blockingantibody, and were inhibited by TLR4 antibody, but, the level of phospho-STAT3 in TLR5-blocking antibody group was significantly lower than the TLR4-blocking antibody group (Fig 7C). These results suggest that flagellin-induced proinflammatory gene expression and STAT3 activation via TLR5 in macrophages.

### Rapamycin downregulates flagellin-induced proinflammatory genes expression in mouse peritoneal macrophages

**Fig 8:**
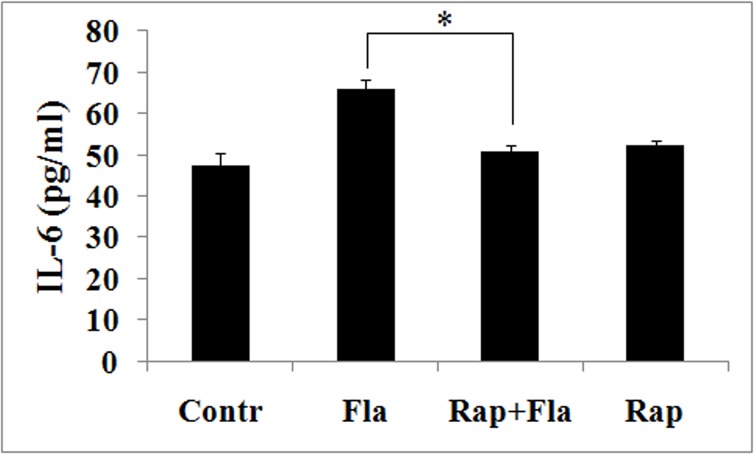
Rapamycin downregulates flagellin-induced IL-6 expression in mouse peritoneal macrophages. Mouse peritoneal macrophages were pretreated with rapamycin (100 nM) for 4 h before being stimulated with 100 ng/ml flagellin for 24 h. Supernatants were assayed for IL-6 by ELISA. flagellin-induced IL-6 expression is inhibited by rapamycin. Experiments were made in triplicate, and data are representative of three separate experiments (mean ± SD). *p< 0.05 compared with flagellin group.

To further examine the role by which mTOR regulates flagellin-induced proinflammatory expression, we measured the effects of rapamycin on IL-6 levels in response to flagellin. Mouse peritoneal macrophages were pretreated with rapamycin before being stimulated with flagellin. IL-6 levels declined significantly due to rapamycin (Fig 8), suggesting that mTOR controls flagellin-induced IL-6 expression in mouse peritoneal macrophages.

## Discussion

Innate immune cells secrete inflammatory cytokines to protect the host from bacterial and viral infection. PI3K is a crucial signaling molecule that is required for macrophages and monocytes during inflammation [37,38], and flagellin activates PI3K through a TLR5-dependent mechanism in human colon epithelial cells [22]. mTOR is a downstream factor of PI3K that mediates the production of inflammatory cytokines by LPS [10,32–35].

PI3K and mTOR participate in the innate immune response and regulate the production of inflammatory cytokines. Yet, the function of PI3K and mTOR in the regulation of flagellin-induced cytokine expression in mouse macrophages is unknown. Flagellin stimulates immune responses by upregulating TNF-α and IL-6 and activating PI3K in epithelial cells and cancer cells [22,39]. In this study, flagellin-induced TNF-α expression was time- and dose-dependently (Fig 1). Thus, we examined the mechanisms in mouse macrophages.

Flagellin governs the proliferation of T cells and cancer cells by upregulating cytokines and chemokines, including TNF-α and IL-6 [40–42], and the role of mTOR signaling in flagellin-induced inflammatory responses is unknown. Our data show that rapamycin downregulates flagellin-induced TNF-α and IL-6 expression (Fig 2C and 2D and Fig 8), moreover, flagellin-induced macrophage proliferation inhibited by rapamycin (Fig 3B). These results are consistent with the function of LY294002 and wortmannin in flagellin-induced TNF-α and IL-6 expression and cell proliferation (Fig 2A and 2B and Fig 3A). Thus, we hypothesize that PI3K/Akt/mTOR controls flagellin-induced cell proliferation by modulating cytokine expression.

The PI3K/Akt pathway activates mTORC1 in response to growth factors [43]. In this study, flagellin stimulated PI3K/Akt/mTOR signaling within 15 min and maintained such activation for at least 24 h (Fig 4A), but these effects were mitigated by LY294002 (Fig 5B). Moreover, LY294002 and rapamycin have the same effect in regulating flagellin-induced TNF-α and IL-6 expression and cell proliferation. These results indicate that PI3K/Akt lies upstream of the mTORC1 pathway in flagellin signaling.

Yu et al. reported that PI3K inhibition enhances flagellin-induced gene expression by prolonging the activation of Erk1/2 in human colon epithelial cells [22]. However, our data demonstrate that LY294002 has no effect on flagellin-induced Erk1/2 activation in mouse macrophages (Fig 5A). Thus, PI3K regulates flagellin-induced cytokines expression but not likely through p44/42 MAPK (Erk1/2) activation.

The NF-κB family of transcription factors are central regulators of immune and inflammatory responses [44], controlling TNF-α and IL-6 expression, and comprises five members in mammals: RELA (also known as p65), c-REL, RELB, NF-κB1 (also known as p105), and NF-κB2 (also known as p100). p65 contains a strong transcriptional activation domain and is responsible for most NF-κB transcriptional activity [35,39]. Inhibitors of NF-κB (IκBs) specifically inhibit NF-κB, associating with and retaining it in the cytoplasm [45]. IκBs are degraded by proteasome, effecting the release NF-κB into the nucleus to initiate transcription [35]. In this study, mTOR signaling pathway regulated NF-κB activation by degrading IκBα in response to flagellin (Fig 6A).

STAT3 has a significant function in blocking apoptosis and keeping cells alive during inflammatory responses, improving the survival and proliferation of myeloma [46]. IL-6 triggers the phosphorylation of STAT3 by JAK1, and the induction of phosphotyrosine705 STAT3 by IL-6 is unaffected by rapamycin [47], suggesting that IL-6-induced STAT3 activation is not regulated by mTORC1. In our study, flagellin induced the phosphorylation of STAT3, which rapamycin inhibited (Fig 6B), indicating that flagellin-induced STAT3 activation is regulated by mTORC1. We hypothesize that flagellin and IL-6 activate STAT3 through disparate signaling pathways.

Bacterial flagellin reconstitutes TLR5-stimulating activity and rapidly induces IL-6 production [1]. Our results showed that flagellin-induced TNF-α and IL-6 expression were blocked by TLR5 antibody and TLR5 siRNA (Fig 7A, 7B and 7D), and flagellin-induced STAT3 activation were declined by TLR5 antibody (Fig 7C). Our results also showed that TLR5 antibody blocked the activation of PI3K/Akt/mTOR signaling stimulated by flagellin (Fig 4B). These findings indicate that TLR5 mediates flagellin-induced TNF-α and IL-6 expression, PI3K/Akt/mTOR signaling activation, and STAT3 activation, these results are proved for the first time in macrophage. In addition, TLR5 recruits the p85 regulatory subunit of PI3K to its cytoplasmic TIR domain through the adaptor molecule MyD88 in response to flagellin [42]. Thus, we propose that TLR5 mediates and transmits flagellin-derived signals to PI3K, for which MyD88 is required.

**Fig 9:**
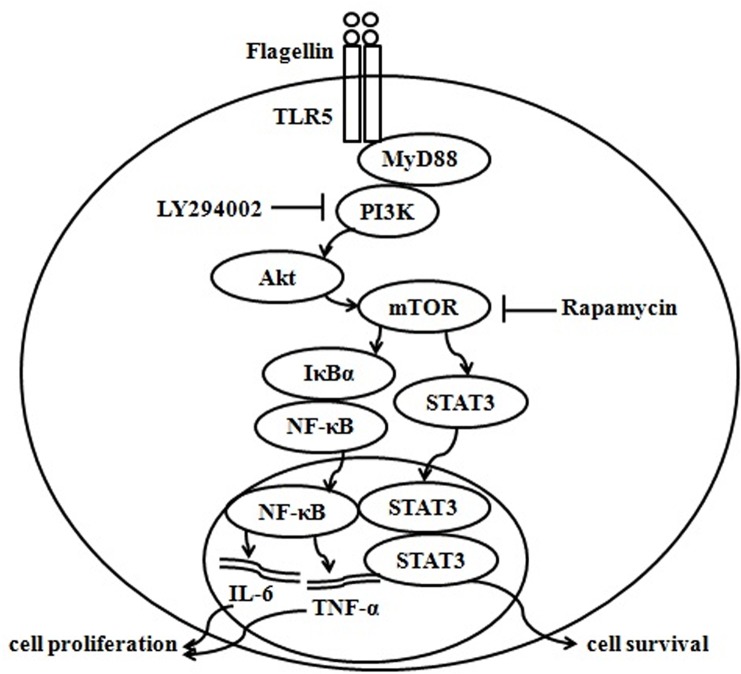
Model of PI3K/Akt/mTORC1 regulation of flagellin-induced TNF-α and IL-6 expression. Based on our studies and other reports, we propose a model in which flagellin binds toTLR5 and transmits its signal to PI3K via MyD88. Activated PI3K stimulatesAkt/mTOR, and mTOR signaling activates NF-κB and STAT3. These factors translocate to the nucleus and enhance TNF-α and IL-6 expression, promoting cell proliferation and survival.

Based on our studies and other reports, we propose a model in which the PI3K/Akt/mTORC1 pathway regulates TNF-α and IL-6 production. Flagellin associates with TLR5 and then transmits its signal to PI3K through MyD88. Activated PI3K stimulates Akt/mTOR, and mTOR signaling then activates NF-κB and STAT3. These translocate into the nucleus, and NF-κB upregulates TNF-α and IL-6 expression, enhancing cell proliferation, and STAT3 promotes cell survival (Fig 9). Although there is no direct evidence that these transcription factors increase TNF-α and IL-6 expression, such a mechanism is possible, depending on their characteristics and functions.

## Supporting Information

S1 Fig Rapamycin decreases flagellin-induced mTORC1 signaling. Ana-1 cells were pretreated with or without rapamycin (100 nM) for 4 h and stimulated with 100 ng/ml flagellin for 24 h. Total proteins were separated by SDS-PAGE and tested using phospho-mTOR (Ser2448), phospho-S6 (Ser240/244), and phospho-4EBP1 (Thr37/46) antibodies. The blots were stripped and probed with mTOR, S6, and 4EBPantibodies. β-actin was used as the loading control. Three separate experiments were performed.
(TIF)
