# Supplementary figures and images for: mTORC1 Regulates Flagellin-Induced Inflammatory Response in Macrophages
Source: PLoS One. 2015 May 5;10(5):e0125910. doi: 10.1371/journal.pone.0125910 (PMC4420466; doi:10.1371/journal.pone.0125910)

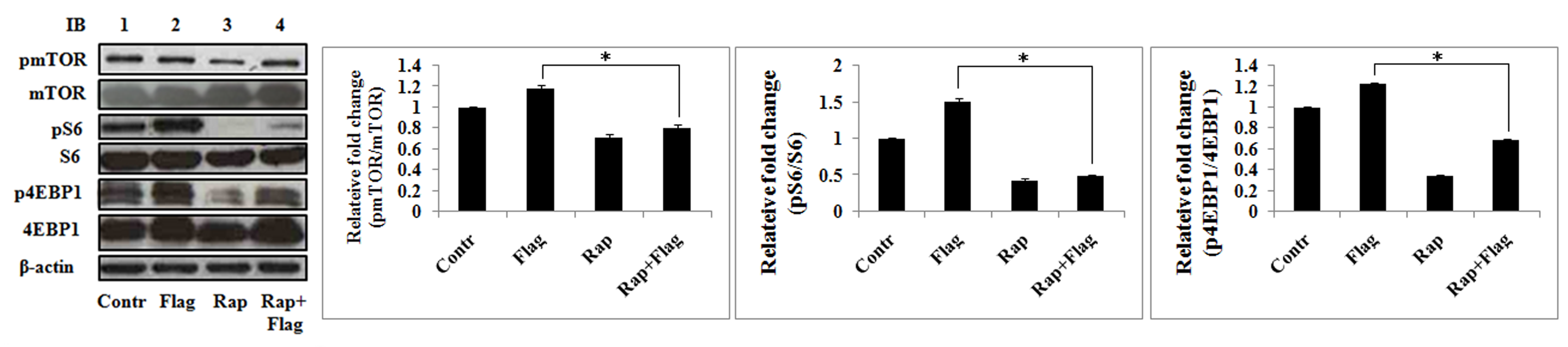

Supplement: S1 Fig — Ana-1 cells were pretreated with or without rapamycin (100 nM) for 4 h and stimulated with 100 ng/ml flagellin for 24 h. Total proteins were separated by SDS-PAGE and tested using phospho-mTOR (Ser2448), phospho-S6 (Ser240/244), and phospho-4EBP1 (Thr37/46) antibodies. The blots were stripped and probed with mTOR, S6, and 4EBPantibodies. β-actin was used as the loading control. Three separate experiments were performed. (TIF) [file pone.0125910.s001.tif]
